# P-130. Comparative study of Brucella native valve Endocarditis and other Culture Positive bacterial native valve infective endocarditis

**DOI:** 10.1093/ofid/ofaf695.357

**Published:** 2026-01-11

**Authors:** Ajithkumar Ittaman, Moideenkutty Gurukkal, Junais koleri, Muna Maslamani

**Affiliations:** HAMAD MEDICAL CORPORATION, Thrissur, Kerala, India; HAMAD MEDICAL CORPORATION, Thrissur, Kerala, India; HAMAD MEDICAL CORPORATION, Thrissur, Kerala, India; HAMAD MEDICAL CORPORATION, Thrissur, Kerala, India

## Abstract

**Background:**

Brucella endocarditis (BE) is a rare but severe form of infective endocarditis (IE), often presenting diagnostic and therapeutic challenges. This study aimed to compare the clinical, microbiological, and outcome parameters of patients with Brucella endocarditis versus age-matched controls with culture-positive infective endocarditis caused by Staphylococcus aureus or Streptococcus species.

**Figure:**
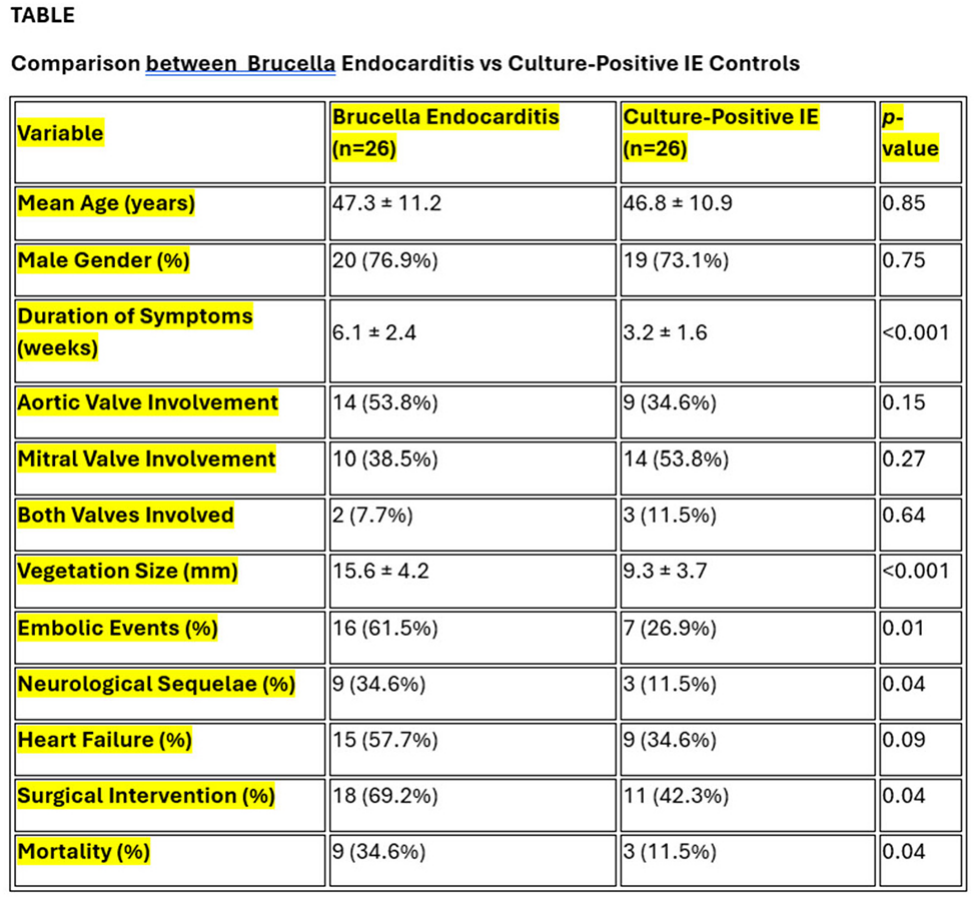

Demographics and observations in Brucella and non brucella bacterial endocarditis groups

**Methods:**

A retrospective comparative study was conducted involving 26 patients with Brucella endocarditis and 26 age- and gender-matched controls with culture-positive IE (S. aureus = 10, Streptococcus spp. = 16). Key variables analyzed included symptom duration, vegetation size, valve involvement, embolic events, neurological sequelae, heart failure incidence, need for surgery, and mortality.

**Results:**

Brucella endocarditis was associated with significantly longer symptom duration, larger vegetations (mean size 15.6 mm vs 9.3 mm, *p* < 0.001), and higher incidence of embolic events (61.5% vs 26.9%, *p* = 0.01). Neurological complications were more common in the Brucella group. The requirement for surgical intervention and the rate of heart failure were higher among Brucella cases. Mortality in the Brucella group was significantly greater (34.6% vs 11.5%, *p* = 0.04).

**Conclusion:**

Brucella endocarditis presents with a more aggressive clinical profile, characterized by larger vegetations, higher embolic and neurological complications, and increased mortality. Early recognition and combined medical-surgical intervention are crucial for improving outcomes.

**Disclosures:**

All Authors: No reported disclosures

